# Potential of Virtual Earth Observation Constellations in Archaeological Research

**DOI:** 10.3390/s19194066

**Published:** 2019-09-20

**Authors:** Athos Agapiou, Dimitrios D. Alexakis, Diofantos G. Hadjimitsis

**Affiliations:** 1 Eratosthenes Research Center, Department of Civil Engineering and Geomatics, Cyprus University of Technology, Saripolou 2-8, Limassol 3036, Cyprus; d.hadjimitsis@cut.ac.cy; 2 Laboratory of Geophysical-Satellite Remote Sensing and Archaeo-Environment, Foundation for Research and Technology, Hellas (F.O.R.T.H.), 74100 Rethymno, Greece; dalexakis@ims.forth.gr

**Keywords:** virtual constellations, remote sensing archaeology, optical sensors, radar sensors, fusion, heritage management

## Abstract

Earth observation sensors continually provide datasets with different spectral and spatial characteristics, while a series of pre- and postprocessing techniques are needed for calibration purposes. Nowadays, a variety of satellite images have become accessible to researchers, while big data cloud platforms allow them to deal with an extensive number of datasets. However, there is still difficulty related to these sensors meeting specific needs and challenges such as those of cultural heritage and supporting archaeological research world-wide. The harmonization and synergistic use of different sensors can be used in order to maximize the impact of earth observation sensors and enhance their benefit to the scientific community. In this direction, the Committee on Earth Observation Satellites (CEOS) has proposed the concept of virtual constellations, which is defined as “a coordinated set of space and/or ground segment capabilities from different partners that focuses on observing a particular parameter or set of parameters of the Earth system”. This paper provides an overview of existing and future earth observation sensors, the various levels of interoperability as proposed by Wulder et al., and presents some preliminary results from the Thessalian plain in Greece using integrated optical and radar Sentinel images. The potential for archaeolandscape studies using virtual constellations is discussed here.

## 1. Introduction

Nowadays, a variety of satellite images with different spatial, temporal, and radiometric resolutions are available due to the recent advancements of earth observation [1]. However, despite the increasing availability of space-borne sensors, research is sometimes restricted by the mismatch observed between the individual sensors’ characteristics in regards to their resolutions. Since each sensor operates in a specific wavelength range, and it is sensitive to specific environmental conditions, the acquisition of all the required information cannot be performed by a single sensor [2].

Optical and radar satellite sensors have been widely applied to support archaeological investigations all around the world. Optical images are used to detect archaeological proxies of subsurface remains [3,4] and to monitor landscapes from natural [5] and anthropogenic hazards, including looting marks [6,7]. Optical image analysis takes advantage of the visible (400–700 nm) and near-infrared part of the spectrum (750–900 nm), while recent studies in the short wavelength infrared part of the spectrum (1500–2300 nm) can also be found [8]. The use of radar spaceborne imagery has shown a significant increase in the last decade, mainly due to the advancement of the spatial and temporal resolution of sensors such as those provided via COSMO-SkyMed, TerraSAR-X, and Sentinel-1 missions [9,10]. Radar images are currently exploited for detecting land movements and landslides in the vicinity of archaeological sites and monuments [11], while the potential use of high-resolution radar sensors for detecting looting areas has been reported in the past [12]. Few publications in regards to the detection of archaeological proxies can be also found [13]. A general overview of the trend observed in the last years in the field of earth observation for archaeological research can be found in the works of [14] and [15].

While it is essential to capitalize on the capacity of existing sensors and to understand potential synergies between them, the concurrent exploitation of radar and spaceborne optical sensors for archaeological research is still limited. In an attempt to expand the scope of space-based Earth system science so as to meet the needs of particular research domains [16], the Committee on Earth Observation Satellites (CEOS) has proposed the concept of virtual, space-based constellations [17]. Virtual constellations are a set of actions aiming to coordinate space and/or ground segment capabilities from different partners related to the Earth system. As [16] argue, “[virtual constellations] are formalized systems, designed to address specific scientific and operational information needs. They involve not only sensors and measurements, but also data policies and archives”. Virtual constellations of planned and existing satellite sensors can help to overcome current limitations in research by combining existing space observations [16].

The integration of space sensors in various research studies can maximize the outcomes and support future research, going thus a step further than the processing of individual datasets. This paper aims to present the concept of virtual earth observation constellations for cultural heritage and archaeological research. The proposed concept is built upon the original work carried out by the Committee on Earth Observation Satellites (CEOS), which formulated the concept of virtual constellations, which was then extended by Wulder et al. [16]. Although fusion and integration analysis of various remote sensing datasets have been reported in the past for archaeological research (see for instance [2,18,19,20,21,22,23], the concept of virtual constellations, proposed by CEOS is more than complementary observations. It is a framework for synergistic and coordinated use of earth observation sensors to increase the data availability, minimizing “unnecessary redundancy and costs” [16]. The opportunities raised by such an approach are discussed in this paper.

## 2. CEOS Virtual Constellations Concept

Virtual constellations are defined as “a coordinated set of space and/or ground segment capabilities from different partners that focus on observing a particular parameter or set of parameters of the Earth system” [17]. Virtual constellations are built upon interagency collaboration and partnerships aiming to “address observational gaps, sustain the routine collection of critical observations, and minimize duplication/overlaps…” [17]. The mission of virtual constellations is to promote the efficient, effective, and comprehensive collection, distribution, improve monitoring, assessment, predictive capabilities, and application of space-based image data of specific domains. Until now, seven virtual constellations have been formed, namely the Atmospheric Composition (AC-VC); the Land Surface Imaging (LSI-VC); the Ocean Colour Radiometry (OCR-VC); the Ocean Surface Topography (OST-VC); the Ocean Surface Vector Wind (OSVW-VC); the Precipitation (P-VC), and the Sea Surface Temperature (SST-VC). Each of these VCs are dealing with a specific area of interest, aiming to implement novel strategies and data collection methods. It should be mentioned that these virtual constellations are based on the close collaboration of the various space agencies dealing with various aspects such as data calibration and validation, merging of satellite and in-situ data, product generation, as well as development and demonstration of new and improved applications. 

**Figure 1:**
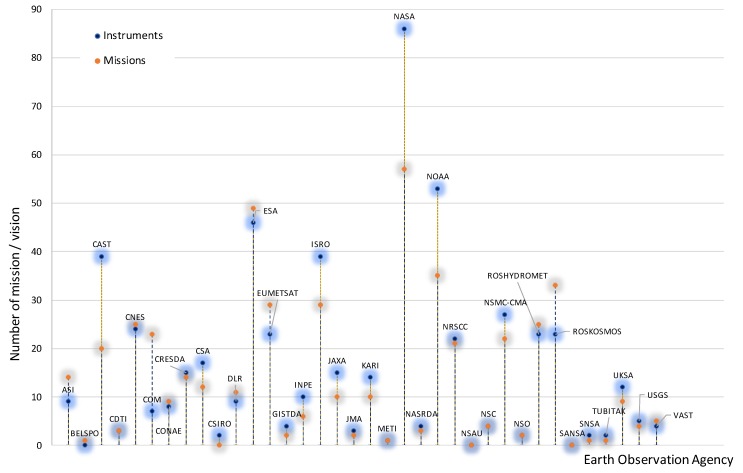
Earth observation operated or approved missions and instruments per agency (data from CEOS Database, [24]).

The need for such collaborations is evident by the variety of missions and instruments available today in the domain of earth observation. Based on the statistics of the CEOS Database [24], more than 35 earth observation agencies are active around the world in more than 25 different countries/regions. These agencies are responsible for nearly 500 earth observation operated or approved missions and up to 560 earth observation instruments (Figure 1).

Although the CEOS dataset is indicative, a closer look into the dataset and putting aside observation missions that are usually out of the scope of the archaeological science (e.g., meteorological sensors), indicates a variety of existing and forthcoming sensors which can provide valuable information for heritage applications, such as those providing optical high-resolution multispectral and hyperspectral images. These high-resolution sensors can acquire submeter resolution optical data. However, the spectral resolution of these sensors is limited to the visible and near-infrared part of the spectrum (see Table 1). A critical attribute of these datasets is access rights and availability of the images. Some high spatial resolution optical and hyperspectral images are currently characterized as “Constrained Access” or “Very Constrained Access”, which prohibits and constrains the scientific exploitation of these datasets. These restrictions, which result in the nonavailability of the satellite images for scientific applications, may limit the potential of exploiting space sensors for better understanding of the environment. Under the prism of data restrictions, the concept of Virtual Constellations is becoming even more imperative: instead of waiting for any future declassification of restricted datasets, the scientific community may focus on the development of new strategies regarding integration of satellite spaceborne datasets. The virtual constellation concept involves the accessibility not only of future datasets, but also for imagery archives.

**Figure 2:**
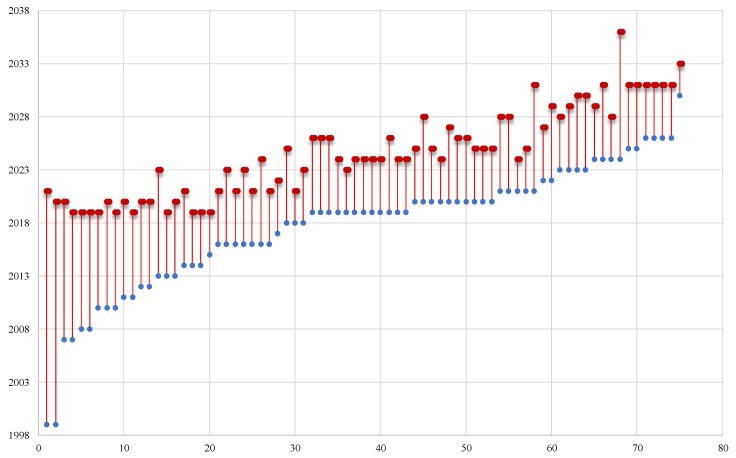
Earth observation sensors (current and future) supporting landscape topography studies (data from CEOS Database, [24]).

As recorded in the CEOS Database [24], the variety of existing and forthcoming sensors for specific categories will continue to grow in the future. For instance, for the domain of landscape topography (for supporting land surface heights measurements, e.g., DEMs), 75 different sensors will provide continuous satellite data until 2036. The list includes 31 active sensors and 44 new sensors which have already been approved (Figure 2). Given these numbers and the trend observed regarding space growth, supported by various national and international space agencies, the concept of the virtual constellation is timely. Synergies between the various space-borne sensors can be developed, including also future earth observation sensors. This close collaboration can eventually fill gaps in terms of the spectral, spatial, and temporal windows. 

Even if initially the concept of virtual constellations was focused on earth observation sensors that share similar spatial, spectral, temporal, and radiometric characteristics, this was later extended by Wulder et al. [16]. In that study [16] virtual constellations “include sensors that are principally incompatible, because they are fundamentally different (for instance active versus passive remote sensing systems), but their combination is necessary and beneficial to achieve a specific monitoring goal.” Additionally, Wulder et al. [16] proposed three application readiness levels (ARL), based on the levels of interoperability of the various datasets, as follow (for more details see [16]): 

**(a)** the first level (ARL-1) combines earth observation sensors whose data are incompatible because the measurements are based on different principles, such as the case of optical and radar sensors;

**(b)** the second level (ARL-2) aims to combine earth observation sensors that share the same principle (e.g., optical sensors) but the sensors have different characteristics (e.g., different spatial/spectral resolutions), and 

**(c)** the third level (ARL-3) aims to combine sensors that share the same principle and share similar spatial and spectral characteristics (e.g., the case of Landsat 5 TM and Landsat 7 ETM+). 

It is evident from the above that the combination of sensors at level 1 (ARL-1) is more complicated and difficult, while processing datasets of level 3 (ARL-3) requires minimal efforts. Examples of the various Application Readiness Levels (ARL-1 to 3) for environmental applications and terrestrial-monitoring can be found in the work of Wulder et al. [16]. 

## 3. Virtual Constellations in Archaeological Research

As mentioned before, in the domain of cultural heritage, earth observation has made a significant contribution, especially towards heritage preservation and management. While the exploitation of single or multisource earth observation datasets has been presented in the past (see for instance [25,26,27]), the synergistic use of such datasets is still limited. Given the spatial, spectral, and temporal resolution limitations of single-space sensors, the virtual constellation concept can improve these characteristics [17,28] to provide more information in a systematic way for monitoring archaeological sites and landscapes. 

The free of charge and open access policy followed by NASA and ESA is a unique opportunity for researchers to explore in depth the synergistic use of space-borne datasets. The high temporal resolution revisits of Sentinel-1 and Sentinel-2 images—provided by the ESA—give the opportunity for researchers to process almost concurrent optical and radar images taken over archaeological areas of interest. In the following section, we present some potential benefits of level ARL-1 at the archaeological landscape of the Thessalian plain in Greece. 

### 3.1. Area of Interest

**Figure 3:**
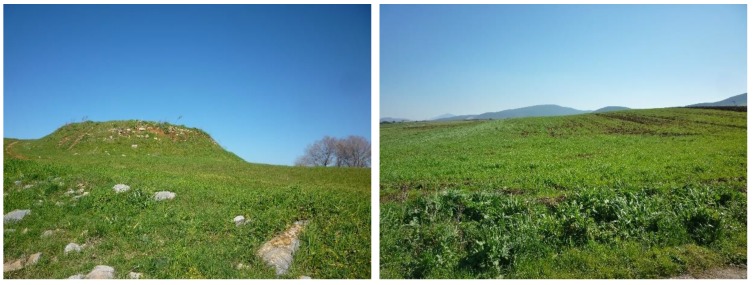
Photo from the magoula Zerelia (**left**) and Almyros II (**right**) (photos from A. Agapiou).

The Thessalian plain comprises a distinct geographical region in central Greece. At the northern part of the plain, there are the Antihasia and Olympus mountains while Orthis and Pindos mountain ranges can be found in the southern and western part of the plain. On the east, the plain ends up towards the Aegean sea. The specific area has attracted the interest of archaeologists since the beginning of the twentieth century. At this region, several Neolithic settlements/tells called magoules were established from the Early Neolithic period until the Bronze Age (6000–3000 BC) (Figure 3). 

The first systematic archaeological studies of the area were performed in 1906, while the first report regarding field surveys results was only published in 1980 [29]. More recently, through the study of Vouzaxakis [29] and Alexakis et al. [30,31], several magoules have been geolocated using global navigation satellite systems (GNSS) and documented with in-situ observations and foot surveys. The overall synthesis of these studies, which cover an area approximately of 13,500 square kilometers, has reported more than 320 archaeological sites. Some of the Neolithic settlements are nowadays visible in the Thessalian plain as low mounds a few meters high, and they mainly consist of loam and mud-based materials. However, due to the agricultural pressure in the area (the Thessalian plain is considered the primary agricultural region of Greece), and the use of heavy agricultural machinery, most the magoules have been flattened, thus making their in-situ detection difficult. 

The discovery of these magoules can, however, be achieved using remote sensing technologies. Indeed, the use of spaceborne optical sensors has been investigated in the past with relative success, indicating cropmarks which are considered as archaeological proxies of these Neolithic settlements of the area [32,33]. These archaeological proxies tend to give a different spectral signature of the crops cultivated on top of the magoules, compared to the rest surrounding the agricultural area as a result of the subsurface remains.

### 3.2. Data Description and Methodology

For the needs of the study, we obtained Sentinel-1 and Sentinel-2 images over the area of interest. The Sentinel images were downloaded using the Sentinel Hub [34] cloud platform, which combines a complete archive of the Sentinel-1 and Sentinel-2 sensors. Specifically, a Sentinel-2 image with an acquisition date of 10th of March 2019 and a Sentinel-1 image with an acquisition date of 8th of March 2019 were downloaded from the Sentinel Hub. The specific acquisition period was selected since this was reported in the literature as the optimum time-window for monitoring cropmarks in the Eastern Mediterranean [35].

**Figure 4:**
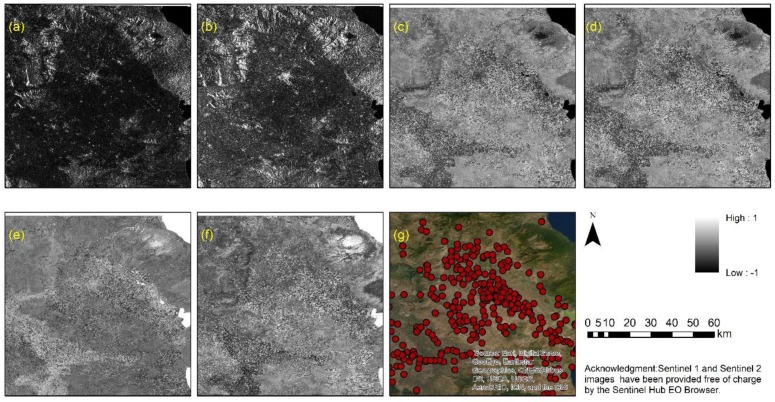
Sentinel-1A over the Thessalian plain acquired at 8 March 2019: (**a**) VH polarization image; (**b**) VV polarization image. Sentinel-2A L2A over the Thessalian plain acquired at 10 March 2019: (**c**) NDVI image; (**d**) NAI image; (**e**) NDWI image and (**f**) NDMI image. The distribution of the Neolithic settlements in the area is shown in (**g**).

The Sentinel Hub provides Sentinel-1 data acquired in interferometric wide swath (IW) mode, with a 250 km swath, processed to Level-1 ground range detected (GRD) at resolution of 10 m, orthorectified at VV intensity (vertical transmit and vertical receive) and VH intensity (vertical transmit and horizontal receive) polarization (Figure 4a,b, respectively). Besides, the Sentinel Hub provides optical Sentinel-2 data processed to two levels: L1C (orthorectified Top-Of-Atmosphere reflectance) and L2A (orthorectified Bottom-Of-Atmosphere reflectance) with spatial resolution of 10 m, 20 m, and 60 m, depending on the wavelength. The spatial resolution of these datasets is considered adequate for the needs of the current study, as the average diameter of the magoules is approximately 100 m.

Based on the reflectance values of the optical Sentinel-2 image, the normalized difference vegetation index (NDVI) [36], the normalized archaeological index (NAI) [37], the normalized difference water index (NDWI) [38], and the normalized difference moisture index (NDMI) [39] have been calculated following Equations (1)–(4). The optical products of Sentinel-2 are shown in Figure 4c–f.

(1) $$NDVI=\frac{\left(\rho_{Band\;8}-\rho_{Band\;4}\right)}{\left(\rho_{Band\;8\;}+\rho_{Band\;4}\right)}$$

(2) $$NAI=\frac{\left(\rho_{Band\;7}-\rho_{Band\;4}\right)}{\left(\rho_{Band\;7}+\rho_{Band\;4}\right)}$$

(3) $$NDWI=\frac{\left(\rho_{Band\;3}-\rho_{Band\;8}\right)}{\left(\rho_{Band\;3}+\rho_{Band\;8}\right)}$$

(4) $$NDMI=\frac{\left(\rho_{Band\;8}-\rho_{Band\;11}\right)}{\left(\rho_{Band\;8}+\rho_{Band\;11}\right)}$$

The two optical and four radar products, namely the VV and VH intensity polarization and the optical indices NDVI, NAI, NDWI, and NDMI, were grouped into a new multiband image for further processing. It should be mentioned that the radiometric range of all bands was within a range scale from −1 to + 1. Then, a random selection of 60% of the geolocated magoules was extracted from the archaeological geo-database. Based on this subset and the multiband image, the properties of each site were estimated, providing the distinctive signature of the site in regards to the VV, VH, NDVI, NAI, NDWI, and NDMI bands. Afterward, a one-class classification processing was followed based on the mean signature of all sites and spectral angle mapper (SAM) as the classifier algorithm. A threshold of 0.05 of the SAM angle was defined. The outcome of this process was a binary classification image with values of 0 and 1. Value 1 indicated areas (pixels) that share similar signature properties as those of the selected sites, while the 0 value indicated unclassified areas.

**Figure 5:**
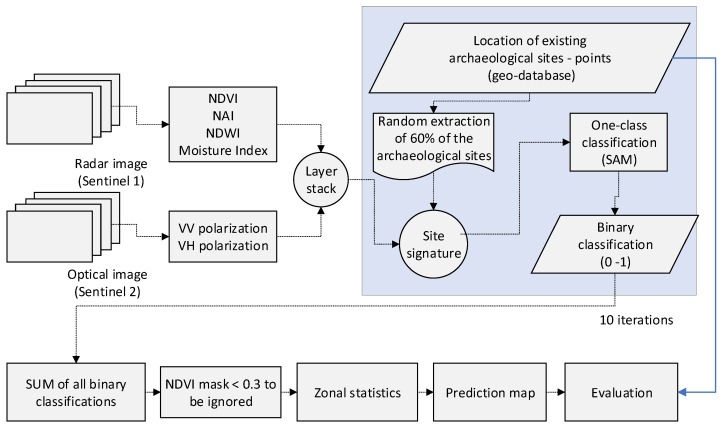
The overall methodology for the prediction map and evaluation at the Thessalian plain.

The random 60% extraction and classification analysis was repeated ten times to examine the robustness of the overall analysis. All binary classifications results were summed up and masked using the NDVI index. The latest was used so as to exclude nonvegetated areas such as urban areas and water bodies (threshold value >0.3). The overall outcomes of the analysis were evaluated and compared using the existing geodatabase (known magoules). The results were quantified using zonal statistics (mean values), while a prediction map was also produced. The methodology diagram is depicted in Figure 5.

### 3.3. Data Analysis

**Figure 6:**
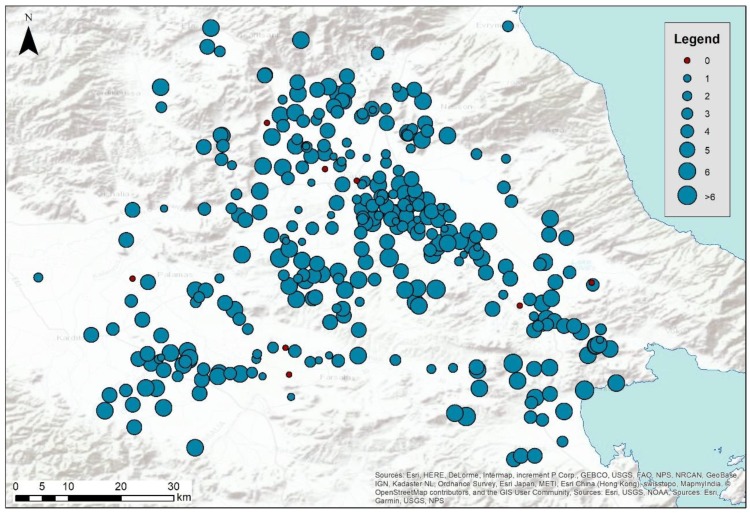
Geographical distribution of the total detections from one-class classification (max = 10: total number of iterations, min = 0: nondetected).

The overall one-class classification rate detection results after the ten iterations are shown in Table 2. The minimum number of detections is zero (nondetected in any of the iterations carried out) while the maximum number of detections is ten (equal to all number of iterations). As shown in the table, only eight sites out of 329 (equal to 2.43%) were nondetected from the classification process. Approximately 15% (52 sites) were detected less than two times, and another 25% of sites (84 sites) were detected less than four times. In total, approximately 44% of the sites were detected less than four times out of the ten of the classification process. In contrast, the rest of the sites (56%) were detected more than six times. The geographical distribution of the detection rate is shown in Figure 6. Red dots indicate the nondetected sites after the ten iterations of the SAM classification process, while the radius of the rest of sites is related to the total number of detections.

**Figure 7:**
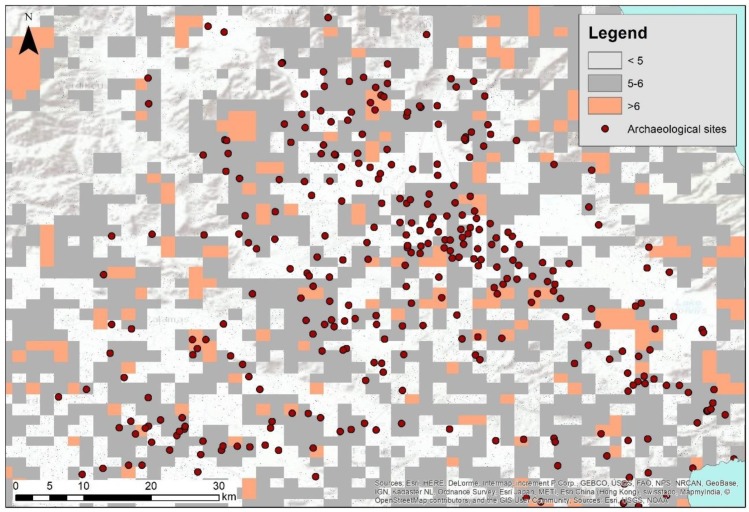
Prediction maps based on the one-class classification.

Based on the outcomes of the one-class classification analysis, a prediction map was generated, as shown in Figure 7. Areas sharing a similar profile with the randomly selected sites were considered as areas with a high likelihood of archaeological evidence more than six times out of ten in the classification process, while grey areas share moderated likelihood values. Given the limited numbers of satellite images used in the study, as well as the extensive area of interest, this prediction map should be taken with great caution and be compared with the existing archaeological visibility of the area. However, like any prediction map in archaeological science, this kind of analysis can support initial steps of landscape research, aiming to visualize the context of an area before any other targeted investigation (e.g., acquisition of high-resolution images, foot surveys, and geophysical prospections) is conducted.

## 4. Discussion

Previous results have demonstrated the potential use of integrated optical and radar satellite images for remote sensing archaeological investigations in the area of Thessaly. While the analysis is based on a limited number of images (one optical and one radar), the overall results show that more of the half-sites (>50%) could be detected following a supervised one-class classification analysis. Of course, difficulties still occur since some sites were not detected, and others were detected only in some of the classification results, which can be explained due to the heterogeneous individual characteristics of the sites.

Although the use of medium resolution freely distributed datasets has the potential to assist towards the detection of new archaeological sites, critical aspects need to be taken into consideration, for instance, the spatial resolution of such datasets. In the case of the Thessalian plain, the use of such images was feasible mainly due to the vast extent of the magoules (100 m diameter). However, the medium resolution does not provide the highest level of accuracy compared to the high-resolution sensors.

However, while high-resolution images with submeter spatial analysis can provide more detail regarding the archaeological area of interest as shown in several examples in the past [40,41], the use of medium freely distributed datasets should not be overlooked. On the contrary, the exploitation of these datasets should be seen as a complementary source of information for supporting large scale archaeological investigations, such as the case of the Thessalian Plain, and are possibly able to unlock new research avenues of future multitemporal archaeological investigations. While the synergistic use of optical and radar images is still in its infancy, it is considered promising [4]. Of course, the virtual constellation concept is not restricted to freely distributed images such as those of Sentinel 1 and Sentinel 2, but it also includes commercial images (either optical or radar), which in combination can support archaeological investigations. Big data earth processing cloud platforms which can support large scale and extensive datasets of observations are considered as essential aspects to support similar future investigations [42,43].

## 5. Conclusions

New developments of earth observation sensors and the development of advanced image processing are some fundamental changes observed in the last years in the field. Due to the variety of sensors available today as well as the variety of space agencies dealing with satellite missions and instruments, the CEOS has proposed a conceptual framework where these could be used together for the benefit of the scientific community by providing harmonized data, filling observation gaps, and improving monitoring, etc. This concept has been lately extended by [16], aiming to include other types of sensors which are incompatible between them as in the case of active and passive space-based sensors.

This framework allows researchers to explore new ways of fusion, better integration, and understanding of the environment that is essential for various scientific domains. This framework could also support heritage management and archaeological prospection, thus maximizing the benefit of space sensors in specific domains. This integration is not an easy task, but it could fill gaps observed using established methods. As the results have shown, difficulties still exist and thus further research is needed. At the same time, efforts for integrating historical data (satellite and aerial), which are essential to better understand landscape changes, are also needed.

Virtual constellation requires the systematic efforts of the scientific community as well as space agency authorities towards the integration of various types of sensors for archaeological research. Therefore, the virtual constellation concept can be the framework for this effort as this has been already implemented with success in other scientific fields. From the experience and work of other ongoing constellations [16], it is clear that the cultural heritage section needs to investigate various approaches for gap analyses and requirement assessments, building upon the existing VCs. The authors will continue to investigate how the synergistic use of radar and optical datasets (level ARL-1) can be further exploited in a more systematic way.

## Figures and Tables

**Table 1:** Details of current and future of earth observation instruments providing high-resolution optical data (source: CEOS Database, [24]).

Instrument Sort and Full Name	Agencies/Country	Instrument Status *	Wavelength	Resolution (m)	Data Access **
AEISS—Advanced Electronic Image Scanning System	KARI/Korea (ASTRIUM/Europe)	O	VIS, NIR	Pan: 0.7 m VNIR: 2.8 m	VCA
AEISS-A—Advanced Electronic Image Scanning System-A	KARI/Korea (ASTRIUM/Europe)	O	VIS, NIR, MWIR	Pan: 0.7 m VNIR: 2.8 m IR: 5.5 m	VCA
AEISS-HR—Advanced Electronic Image Scanning System-High Resolution	KARI/Korea (KAI)	F	VIS, NIR	Pan: 0.5 m, VNIR: 2 m	VCA
APAN—Advanced PAN	ISRO/India	F	VIS	1.25	CA
ASTER—Advanced Spaceborne Thermal Emission and Reflection Radiometer	METI/India (NASA)/USA	O	VIS, NIR, SWIR, TIR	VNIR: 15 m SWIR: 30 m TIR: 90 m	OA
ATCOR—Atmospheric correction	ISRO/India	F	VIS, NIR	240 m	CA
CARBONITE-1 Imager	UKSA/UK	O	VIS	1.5 m	VCA
CARBONITE-2 Imager	UKSA/UK	O	VIS	1.2 m	VCA
CCD (HJ)	CAST/China	O	VIS, NIR	30 m	OA
Event Imaging Spectrometer from GEO (GeoCape)	NASA/USA	F	VIS	250 m	OA
Geoton-L1	ROSKOSMOS/Russia	O	VIS, NIR	1 m; 3 m	CA
High Resolution Optical Sensor	KARI/Korea	F	VIS, NIR	1m	VCA
HiRI—High-Resolution Imager	CNES/France	O	VIS, NIR	0.70 m	OA
LISS-III—Linear Imaging Self Scanner III (Resourcesat)	ISRO/India	O	VIS, NIR, SWIR	23.5 m	OA
LISS-IV	ISRO/India	O	VIS, NIR	5.8 m	CA
LISS-V—Linear Imaging Self Scanner – IV	ISRO/India	F	VIS	2.5	CA
MSC—Multi-Spectral Camera	KARI/Korea	O	VIS, NIR	Pan: 1 m VIS-NIR: 4 m	VCA
MSI—Multi Spectral Imager	DLR/Germany	O	VIS, NIR	6.5 m	OA
MSI (Sentinel-2)—Multi-Spectral Instrument	ESA/Europe (COM/Europe)	O	VIS, SWIR	10/20 m	OA
MSS—Multispectral imaging system	ROSKOSMOS/Russia (ROSHYDROMET/Russia)	O	VIS, NIR	12 m	CA
MUX (SJ-9A)—Multispectral CCD Camera	CRESDA/China	O	VIS, NIR	10 m	OA
MX (HRSAT)—Multispectral HR VNIR	ISRO/India	F	VIS	1.9 m	CA
NigeriaSat 2 Remote Sensing—Med and High Res	NASRDA/Nigeria	O	VIS, NIR	Pan: 2.5 m MS: 5/32 m	
OEK VR—Multispectral optoelectronic high-resolution module	ROSKOSMOS/Russia	F	VIS, NIR	Pan: 0.4 m MS: 1.6 m	CA
PAN (BJ-2)—Panchromatic Imager	NRSCC/China	O	VIS	1 m	
PAN (Cartosat-1)—Panchromatic Camera	ISRO/India	O	VIS	2.5 m	CA
PAN (Cartosat-2)—Panchromatic Camera	ISRO/India	O	VIS	1 m	VCA
PAN (Cartosat-2A/2B)—Panchromatic Camera	ISRO/India	O	VIS	1 m	VCA
PAN (Cartosat-2E)—Panchromatic Camera	ISRO/India	O	VIS	0.65 m	VCA
PAN (Cartosat-3)—Panchromatic sensor	ISRO/India	F	VIS	0.25 m	VCA
PAN (CBERS)—Panchromatic and Multispectral Imager	CAST/China	O	VIS, NIR	Pan: 5 m MS: 10 m	OA
GF-1/PMS -Panchromatic and multispectral imager	CRESDA/China	O	VIS, NIR	Pan: 5 m MS: 10 m	OA
GF-2/PMS—Panchromatic and multispectral imager	CRESDA/China	O	VIS, NIR	Pan: 5 m MS: 10 m	OA
PAN (GISTDA)—Panchromatic imager	GISTDA/Thailand	O	VIS	2 m	CA
PAN (HRSAT)—Panchromatic Camera	ISRO/India	F	VIS	1 m	CA
PAN (SJ-9A)—Panchromatic and multispectral imager	CRESDA/China	O	VIS, NIR	2.5 m	OA
PAN (ZY-1-02C)—Panchromatic and multispectral imager	CRESDA/China	O	VIS, NIR	Pan: 5 m MS: 10 m	VCA
PAN CAMERA—Panchromatic Camera	ASI/Italy	F	VIS	5 m	CA
PAN THEOS-2 (GISTDA)—Panchromatic imager THEOS-2	GISTDA/Thailand	O	VIS	2 m	CA
PAN+MS (RGB+NIR)—Ingenio PAN+MS	CDTI/Spain (ESA/Europe)	F	VIS, NIR	Pan: 2.5 m MS: 10 m	CA
PSS—Panchromatic imaging system	ROSKOSMOS/Russia (ROSHYDROMET/Russia)	O	VIS, NIR	2.5 m	CA
VHRI-100—Very High-Resolution Imager 100	UKSA/UK	O	VIS, NIR	Pan: 1.0 m MS: 4.0 m	CA
WPM—Wide Swath Panchromatic and Multispectral Camera	CAST/China	F	VIS, NIR	Pan: 2 m MS: 8 m	OA

* O: Operational, F: Future

** VCA: Very Constrained Access; CA: Constrained Access; OA: Open Access

**Table 2:** Overall results from the evaluation report.

Description	Total Number	%	Cumulative %
Nondetected sites	8	2.43	2.43
Sites detected less than 2 times	52	15.81	18.24
Sites detected less than 4 times	84	25.53	43.77
Sites detected less than 6 times	179	54.41	98.18
Sites detected more than 6 times	6	1.82	100.00
